# A new parametric model to assess delay and compression of mortality

**DOI:** 10.1186/s12963-016-0113-1

**Published:** 2016-12-01

**Authors:** Joop de Beer, Fanny Janssen

**Affiliations:** 1 Netherlands Interdisciplinary Demographic Institute/University of Groningen, PO Box 11650, The Hague, 2502 AR The Netherlands; 2 Population Research Centre, Faculty of Spatial Sciences, The Netherlands & Netherlands Interdisciplinary Demographic Institute, University of Groningen, The Hague, The Netherlands

**Keywords:** Probability of death, Mortality age schedule, Rectangularization, Compression, Age at death distribution, Life expectancy, Decomposition

## Abstract

**Background:**

A decrease in mortality across all ages causes a shift of the age pattern of mortality, or mortality delay, while differences in the rate of decrease across ages cause a change in the shape of the age-at-death distribution, mortality compression or expansion. Evidence exists for both compression and delay of mortality. Existing parametric models to describe the full age pattern of mortality are not able to capture mortality delay versus mortality compression. More recent models that assess delay versus compression mostly focused on the adult or old ages alone and did not distinguish mortality compression below and above the modal age at death, although they represent different mechanisms.

**Methods:**

This paper presents a new parametric model that describes the full age pattern of mortality and assesses compression – at different stages of life – and delay of mortality: the CoDe model. The model includes 10 parameters, of which five are constant over time. The five time-varying parameters reflect delay of mortality and compression of mortality in infancy, adolescence, young adulthood, late adulthood, and old age. The model describes infant and background mortality by two simple functions, uses a mixed logistic model with different slopes in adult, middle, and old age, and includes the modal age at death as a parameter to account for the delay in mortality.

**Results:**

Applying the CoDe model to age-specific probabilities of death for Japanese, French, American, and Danish men and women between 1950 and 2010 showed a very good fit of the full age pattern of mortality. Delay of mortality explained about two-thirds of the increase in life expectancy at birth, whereas compression of mortality due to mortality declines in young age explained about one-third. No strong compression of mortality in late adulthood age was observed. Mortality compression in old age has had a small negative impact on life expectancy.

**Conclusions:**

The CoDe model proved a valid instrument for describing the full age pattern of mortality and for disentangling the effects of mortality delay and compression – at different stages of life – on the increase in life expectancy.

## Background

Since the Second World War mortality has decreased strongly across developed countries, resulting in an enormous increase in life expectancy. Changes in mortality at different ages have contributed to the change in overall mortality and in life expectancy. We can distinguish two types of change: (i) compression, caused by differences in the rate of decrease across ages which changes the shape of the age pattern of mortality [1], and (ii) delay, i.e., a decrease in mortality across all ages which does not change the age pattern of mortality [2]. For the post-war period evidence exists for both compression [3–10] and delay of mortality, also referred to as the shifting-mortality regime [6, 7, 11–15]. The relative roles of the two processes in the mortality trend are important for the future development of life expectancy. If only mortality compression occurs, we would be approaching a limit to life expectancy. If, however, delay in aging occurs, a limit to life expectancy is unlikely for the near future. Also, the relative roles of delay versus compression give information on the main determinants of the increase in life expectancy.

The main determinants of mortality delay, indicated by an increase in the modal age at death, are increased prosperity and improvements in medicine [2]. Better living and working conditions make people healthier, while improvement of public health and medical treatment prevent and mitigate illness. Changes in the shape of the age-at-death distribution have many different causes. The age distribution becomes more compressed if mortality at young ages decreases *more* strongly than at ages around the modal age at death and if mortality at the oldest ages decreases *less* strongly than around the modal ages. Both types of compression are caused by different developments. Compression before the modal age at death reflects a decline in premature deaths, i.e., a shift of deaths from young ages toward ages around the modal age at death. Infant mortality has decreased due to prevention and better treatment of infectious disease and improvement of maternal and child health care. Mortality at young adult ages has decreased due to safety measures leading to fewer traffic accidents. Mortality in middle age has decreased due to an improvement in the treatment of cardiovascular disease and a decline in the prevalence of smoking. Compression before the modal age has led to an acceleration in the increase in life expectancy. In contrast, compression above the modal age has a downward effect on the rate of increase in life expectancy, since it implies that mortality at advanced age decreases less strongly than at ages around the modal age at death. This may be due to limited progress in the treatment of diseases at advanced age, such as dementia. Since compression below and above the modal age at death have different causes and different effects on life expectancy, it is important to distinguish both types of compression.

Previous parametric models to describe the full age pattern of mortality were not able to capture mortality delay versus mortality compression [16, 17]. They date back to over 30 years ago, included many parameters that were difficult to interpret, and did not include recent advancements in describing mortality at older ages [4, 18]. More recent models that specifically included mortality delay focused on adult ages alone [12, 19]. Also, the few previous attempts to assess delay versus compression did not distinguish mortality compression below and above the modal age at death [20, 21].

This paper introduces a new parameterized model of the full age pattern of mortality which integrates recent new insights and allows us to disentangle the effects of delay and compression of mortality – at different stages of life – on changes in life expectancy. We call the new model the CoDe model as it models *com*pression and *de*lay of mortality.

The CoDe model includes five additive components describing mortality in infancy, adolescence, young adult age, late adulthood, and advanced age. The model includes 10 parameters, but only five parameters vary over time. Changes in the values of the five interpretable time-varying parameters reflect mortality delay and compression of mortality at different stages in life and allow us to calculate the effects of these changes on the age distribution and on life expectancy.

## Methods

After a review of earlier parametric mortality models, and an earlier attempt to assess the contribution of mortality delay and compression to changes in life expectancy, we set out the CoDe model using mortality among French women in 1950 and 2010 as an example. In addition, we apply the model to probabilities of death between 1950 and 2010 for women and men in four low-mortality countries: Japan, France, the US, and Denmark.

For this purpose, we obtained sex- and age-specific unsmoothed data on annual death rates from the Human Mortality Database (HMD) [22] from age 0 to 100. We estimated probabilities of death *q(x)* from the death rates and calculated life table estimates of the age-at-death distribution *d(x)* and the survivor curve *l(x)* using the same life table techniques as used by the HMD [23].

### Review of parametric mortality models

The age pattern of mortality has a similar shape in most countries. Mortality is high in infancy, decreases strongly during early childhood, increases sharply in teenage years (this is often called the “accident hump”), increases exponentially during adulthood, and its rate of increase levels off in old age [16, 17, 24–27]. Parametric models are aimed to represent this age pattern by a mathematical model including a limited number of parameters. Parametric models are used to smooth age-specific death rates, to make comparisons of mortality across countries, and to make forecasts based on an analysis of changes in the values of the parameters [26].

In 1825, Gompertz [28] proposed the first “law of mortality” describing the exponential increase of mortality in adult age. Even though the Gompertz model provides an accurate fit for most adult ages, the model tends to underestimate mortality at young adult ages and to overestimate mortality at the oldest ages [12, 29]. To address the underestimation of mortality at young adult ages, Makeham [30] added a constant in 1860. This constant is usually referred to as background or non-senescent mortality which does not vary with age, e.g., deaths caused by accidents and certain infections [12, 31–33]. Background mortality can change with time but is assumed constant above age 25 or so [12]. The Gompertz-Makeham model, however, does not describe the decline of mortality during infancy and childhood [34].

The first model to describe the full age pattern of mortality was developed by Thiele in 1871 [24]. Around 1980, Siler [16] and Heligman & Pollard [17] developed their models to describe mortality during the entire age span. In the 1990s, Kostaki [35] slightly adjusted the Heligman-Pollard model, and Rogers & Little [36] developed another model. Most of these models consist of additive components representing mortality at subsequent stages of life. Most often models include three components to describe death in (i) infancy and childhood, (ii) adulthood, and (iii) old age [26].

To describe the mortality decline during infancy and childhood, Siler [16] and Rogers & Little [36] used an exponential curve based on the model proposed by Thiele [24, 26, 27]. It proved, however, that an exponential model does not describe very accurately the decline in mortality in young childhood. Heligman and Pollard [17] therefore proposed a double exponential function that provides a more accurate description of the decline in mortality during childhood. The Heligman-Pollard specification includes three parameters instead of the two parameters used by Thiele [24], Siler [16], and Rogers and Little [36]. The values of these three parameters are highly correlated, which makes it difficult to estimate and interpret changes in those values [37].

To describe the excess mortality in early adulthood Thiele [24] proposed a Gaussian function. Alternatively, Heligman and Pollard [17] proposed an asymmetric function for this elevated mortality. Both specifications include three parameters. Kostaki [35] even added a fourth parameter to the Heligman-Pollard function to improve the fit. Rogers and Little [36] specified a double exponential function including four parameters.

Thus each of these models requires relatively many parameters to describe mortality in early adulthood. Furthermore, these specifications assume a decline in mortality after early adulthood. This implies that these models do not include background mortality as suggested by Makeham [30]. In contrast, the Siler [16] model includes background mortality but ignores the “hump” at early adulthood.

For adult mortality, the Thiele, Siler, Rogers-Little, and Heligman-Pollard models followed the idea of an exponential increase as described by the Gompertz model and therefore include an exponential term to describe the increase in mortality in adult age.

For mortality at ages 80 and above, it was observed, however, that the rate of mortality increase slows down [4]. Since the Heligman-Pollard model describes the odds ratio of the probability of dying rather than the death probability itself, this model describes levelling off of the increase in mortality at the oldest ages, in contrast with the other three models. Alternatively, the overestimation of mortality at the oldest ages can be addressed by using a logistic model rather than the Gompertz model [4, 18, 38]. Until advanced age the difference between the Gompertz and a logistic model is ignorable. While the Gompertz model has no limit at the oldest ages, the logistic model is capable of describing the levelling off of the increase in mortality at the oldest ages [4, 12]. Beard [39], Horiuchi and Wilmoth [32], and Thatcher et al. [38] examined complex logistic models with additional parameters, but Thatcher [4] and Thatcher et al. [38] conclude that a simple, robust logistic model, the so-called Kannisto [18] model, including only two parameters, provides an excellent fit to mortality rates at older ages. The Kannisto model is also currently used to smooth mortality data included in the Human Mortality Database at the oldest ages [23]. However, it has not yet been incorporated in a mortality model that includes all ages.

In all of the above models, a change in mortality is interpreted as a decline in death rates. Recently, Bongaarts and Feeney [40] and Bongaarts [12] note that instead of interpreting mortality as falling, the schedule of the death rate can be viewed as shifting to higher ages over time. They describe changes in death rates by the so-called shifting logistic model. This model implies that death rates decline due to a shift in the age schedule rather than due to a change in the slope of death rates which changes the shape of the age schedule. Focusing on adult mortality, Bongaarts and Feeney [40] show that the shifting logistic model describes changes over time with only one time-varying parameter. However, Bongaarts [12] admits that there is some loss in the goodness of fit.

### Assessing the effects of compression and delay of mortality

Only very recently, a parametric mortality model has been utilized to decompose changes in life expectancy into the effects of compression and delay of mortality [21]. To do so, Bergeron-Boucher et al. [21] used the Siler model to model the age pattern of mortality, including three terms: one term describes infant mortality, another term is a constant describing background mortality, and the third term is a Gompertz function describing the exponential increase of mortality in adult and old age. The latter term includes the modal age at death as parameter, since Horiuchi et al. [19] showed that including the modal age at death in the Gompertz function model takes account of shifts of the mortality age schedule. The Siler model used by Bergeron-Boucher et al. [21] is

1 $$ m(x)={a}_1{e}^{-{a}_2x}+c+b{e}^{b\left(x-M\right)} $$

where *m(x)* = death rate at age *x*; *a*_*1*_ determines level of infant mortality at age 0 and *a*_*2*_ determines the decline of mortality in early childhood; *c* = background mortality which does not change with age, *M* = modal age at death, *b* is the rate of change in adult and senescent mortality. A high value of *b* implies low death probabilities at young adult ages and high death probabilities at the oldest ages. Thus an increase in *b* results in compression of mortality around the modal age [41]. Since the parameter *b* affects both compression in adult and old age, this model cannot distinguish between compression below and above the modal age at death. This model assumes that compression in adult age always results in compression in advanced age as well.

**Fig. 1:**
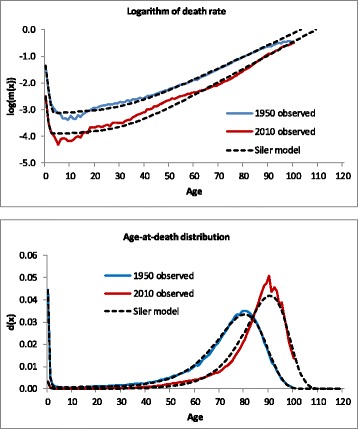
Logarithm of death rate and age-at-death distribution, French women, 1950 and 2010, observed and fitted by the Siler model

Figure 1 shows the fit of the Siler model to the logarithm of the death rate log(*m(x)*) and the age-at-death distribution *d(x)* for French women. In addition, the figure shows extrapolations based on the fitted model until age 120. The figure shows that the Siler model provides a reasonable fit for 1950 but that the fit for 2010 is not very accurate. The upper graph shows that the model underestimates the death rate between ages 20 and 60, and the lower graph shows that the model underestimates deaths around the modal age. Thus the model underestimates compression of mortality in 2010.

### The CoDe model

We propose a new model that (1) describes the full age pattern of mortality, (2) provides a better fit to the data, and (3) allows us to distinguish mortality compression in young, adult, and advanced ages: the CoDe model.

The CoDe model (see formula 2 below) includes 10 parameters, of which five vary over time. The five interpretable time-varying parameters reflect delay of mortality and changes in mortality in infancy, adolescence, middle age, and old age. The model describes young age mortality by a simple function, includes a term that describes both the teenage “hump” and the level of background mortality, uses a mixed logistic model with different slopes in adult, middle, and advanced age, and includes the modal age at death as a parameter to account for the delay in mortality. These different elements are described in more detail below.

To model mortality at the youngest ages, instead of Heligman and Pollard [17], who specified a double exponential term ${A}^{x+{B}^c}$, we propose a simple function assuming an inverse relationship of mortality with age: *A*/(*x* + *B*). *A* reflects the level of infant mortality and *B* affects the rate of decrease of mortality in childhood. Since the effect of changes in the value of *B* over time turns out to be relatively small, changes in mortality at the youngest ages can mainly be attributed to changes in the value of *A*. This makes interpretation of this term straightforward: lower infant and childhood mortality corresponds with a lower value of *A*. Figures 9 and 10 in Appendix 1 show that the fit of the CoDe model at ages up to five is similar to the fit of the Heligman-Pollard model.

In teenage years death probabilities increase relatively strongly, followed by a leveling off at young adult ages. This is usually called the “accident hump” of mortality. Heligman and Pollard [17] describe this hump by an exponential function including three parameters: *Dexp*[−*E*(*logx* − *logF*)^2^]; but this does not include background mortality [12, 30], i.e., the part of mortality in adult age that does not vary with age. The Siler model [16, 21] includes background mortality but does not include the hump. We propose an alternative specification that describes the sharp increase in mortality at teenage years (the hump) and assumes a constant level in adult age, and thus accounts for background mortality as well. This pattern can be described by a simple logistic function: $\frac{a{e}^{\left(x-m\right)}}{1+{e}^{\left(x-m\right)}}$, where *m* determines at which teenage ages mortality increases most strongly, and *a* is the level of background mortality. Fitting the model for women and men in Japan, France, the US, and Denmark for both 1950 and 2010 shows that *m* = 16 provides an accurate fit. This means that in the CoDe model only one parameter is sufficient to describe the excess mortality in adolescence. Figure 11 in Appendix 2 shows that for mortality in adolescence the fit of the CoDe model is better than that of the Heligman-Pollard model.

In line with the findings by Kannisto [18] and Thatcher [4], discussed above, we include a logistic rather than a Gompertz function to model mortality in adult and old age. In order to be able to make a distinction between compression in adult age and in advanced age we introduce separate logistic terms to describe mortality before and above the modal age at death.

Fitting a model that combines the aforementioned components to data for Japan, France, the US, and Denmark in the period 1950–2010 showed that the model provides an accurate fit for early years in the period, but that for more recent years the fit is not perfect around middle age. Therefore we subdivide mortality before the modal age at death into mortality in young adulthood and mortality in late adulthood.

The resulting CoDe model thus includes five additive terms representing mortality at subsequent stages of life. The first two terms are used to model mortality at young ages and mortality in adolescence. The remaining three terms are included to make a distinction between mortality in early adulthood, late adulthood, and old age, respectively.

The CoDe model is formulated by:

2 $$ \begin{array}{c}\hfill q(x)=\frac{A}{x+B}+\frac{a{e}^{\left(x-16\right)}}{1+{e}^{\left(x-16\right)}}+I\left(x\le M-h\right)\left[\frac{b_1{e}^{b_1\left(x-M\right)}}{1+\frac{b_1}{g}{e}^{b_1\left(x-M\right)}}\right]\hfill \\ {}\hfill +I\left(M-h<x\le M\right)\left[\frac{b_2{e}^{b_2\left(x-M\right)}}{1+\frac{b_2}{g}{e}^{b_2\left(x-M\right)}}+{c}_1\right]+I\left(x>M\right)\left[\frac{b_3{e}^{b_3\left(x-M\right)}}{1+\frac{b_3}{g}{e}^{b_3\left(x-M\right)}}+{c}_2\right]\ \hfill \end{array} $$

where *I(.)* is an indicator function; *b*_*1*_ determines the slope of the curve of death probabilities before age *M - h*, *b*_*2*_ between ages *M - h* and *M,* and *b*_*3*_ above the modal age at death *M*; and *c*_*1*_ and *c*_*2*_ are constants that are introduced to avoid “jumps” in the fitted values at ages *M - h* and *M*. That is, we require that the values of the third and fourth terms on the right hand side of (2) be equal at age *x* = *M - h* and the fourth and fifth terms be equal at age *M*. This implies that ${c}_1=\frac{b_1{e}^{b_1\left(-h\right)}}{1+\frac{b_1}{g}{e}^{b_1\left(-h\right)}}-\frac{b_2{e}^{b_2\left(-h\right)}}{1+\frac{b_2}{g}{e}^{b_2\left(-h\right)}}$ and ${c}_2=\frac{b_2}{1+\frac{b_2}{g}}+{c}_1-\frac{b_3}{1+\frac{b_3}{g}}$.

In order to make the estimates of the parameters comparable across years and across countries we assume the same value of *h* for all countries in all years between 1950 and 2010. Since we want to divide adulthood into three parts (young and late adulthood and old age), we fix *h* at 30 years. As the average value of the modal age at death is around 80 years, choosing *h* = 30 implies that *b*_*1*_ affects mortality below around 50 years (young adulthood), *b*_*2*_ affects mortality between around 50 and 80 years (late adulthood), and *b*_*3*_ affects mortality above around 80 years (old age). We also assume the same value of *g* across countries and years. Gampe [42] who studied mortality beyond age 110 in detail – based on numerous countries for which the data are regarded reliable – estimated that the death probabilities above age 110 are flat at a value of 0.5. In contrast, the HMD assumes an upper bound of 1 for the death rates [23] which corresponds with an upper bound of 0.7 for death probabilities. Appendix 8 shows that estimating the parameters of the CoDe mortality model, it turns out that *g* = 0.7 provides a slightly better fit for the four countries in this study in the period 1950–2010 for ages up to 100 as compared to *g* = 0.5. Therefore, we fixed the value of *g* at 0.7. Sensitivity analyses in Appendix 8 show that our results on the extent of compression and delay would not differ much if we had assumed *g* = 0.5.

**Fig. 2:**
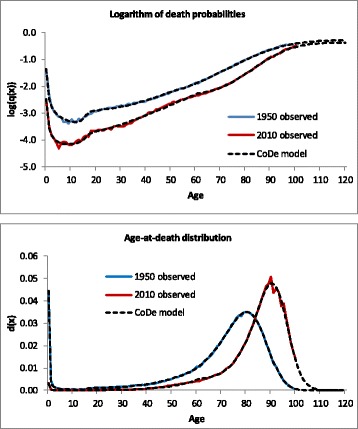
Logarithm of death probabilities and age-at-death distribution, French women, 1950 and 2010, observed and fitted by the CoDe model

Figure 2 shows that the CoDe model provides a good fit to the age pattern of the probabilities of death as well as the age-at-death distribution for French women in both 1950 and 2010. Appendix 3 shows – again for French women – the contribution of the separate terms of equation (2) to the fit of the model, illustrating how compared to the logistic model adding terms for mortality in infancy, adolescence, young adulthood, and late adulthood improves the fit of the model for both death probabilities and the age-at-death distribution. Overall, the fit of the CoDe model is more accurate than the Siler model (see formula (1)), as can be viewed when comparing Fig. 2 to Fig. 1. In addition, however, the fit is also better than the Heligman-Pollard model. See Fig. 14 and Table 2 in Appendix 4.

Fitting the CoDe model to data for French women for all years in the period 1950–2010 shows that while the value of *b*_*1*_ has been constant during this period, the values of *b*_*2*_ and *b*_*3*_ have increased (see Fig. 6). This explains why a simple logistic model provides an accurate fit for 1950, but not for 2010. Thus a model including one slope parameter [12, 40] underestimates the compression of mortality between 1950 and 2010. We avoid this inaccuracy by introducing the parameters *b*_*2*_ and *b*_*3*_ which turn out to change over time.

The CoDe model can be used to assess the effects of changes in mortality at young and old ages on the age pattern of mortality and the effect of delay of mortality on the overall level of mortality. Since *B* is included in the denominator of the first term of model (2), changes in its value have only minor impact on life expectancy compared with changes in the value of *A*. For example, if we multiply the value of *A* for French women in 2010 by 10, life expectancy at birth would decrease by 3 years, while if we multiply the value of *B* by 10, life expectancy would increase by 0.3 years only. Figure 6 shows that even though the level of *b*_*1*_ varies across countries, its value hardly changes across time. This implies that changes in the death probabilities and in the age-at-death distribution can be explained almost solely by changes in the values of five parameters: *A* (infant mortality), *a* (background mortality), *M* (modal age at death), *b*_*2*_ (mortality in late adulthood), and *b*_*3*_ (mortality in old age, i.e., above the modal age).

A decline in *A* results in compression of mortality, as a decrease in deaths in infancy results in more deaths in adult or older age. A decline in *a* (decline in background mortality) which affects mortality in young ages relatively more strongly than in old age results in compression of mortality. High values in *b*_*1*_ and *b*_*2*_ result in compression of mortality before the modal age at death as they result in fewer deaths at young adult and older adult ages, respectively, and more deaths around the modal age at death. A high value of *b*_*3*_ results in a strong increase of probabilities of death in advanced age and thus more deaths around the modal age at death and fewer deaths at the oldest ages. An increase in the modal age at death *M* results in a shift of the curves described by the latter three terms to older ages and thus in a delay of adult mortality.

Note that we fit the model to death probabilities rather than to age-specific death rates. One advantage of using death probabilities rather than death rates is that probabilities are easy to interpret and probabilities rather than rates are used for projections and for the calculation of life expectancy using a life table [43, 44]. However, as the age pattern of death probabilities and rates are very similar, our results and conclusions would have been similar if we had modeled rates rather than probabilities.

Note as well that since our model is aimed to describe the entire age pattern of mortality as accurately as possible, we estimate the parameters by minimizing the mean squared error (MSE) in both *log(q(x)*) and *d(x).* The MSE of *log(q(x))* gives relatively high weight to errors at young ages, while the MSE of *d(x)* gives high weight to errors around the modal age at death. We calculate absolute errors for *d(x)* and *log(q(x))*. Note that calculating errors in the logarithm of *q(x)* comes down to estimating relative errors for death probabilities. We apply weighted least squares by using the reciprocal of the variances as weights to minimize the weighted MSE (MSE[log*q(x)*]/var[log*q(x)*] + MSE[*d(x)*]/var[*d(x)*])/2. Note that this implies that we calculate the average *R* ^*2*^ for *logq(x)* and *d(x)*, as *R* ^*2*^ *= 1 − MSE/var*, and thus that we maximize average *R* ^*2*^ *.*

## Results

We estimate the parameters of the CoDe model by fitting the model to probabilities of death in the years 1950 to 2010 for four low-mortality countries: Japan, France, the US, and Denmark. These four low-mortality countries have shown different trends in life expectancy during the last 60 years. In 1950 life expectancy at birth of American and Danish women was 10 years higher than for Japanese women, whereas in 2010 Japanese women had a 5-year higher life expectancy than American and Danish women. Since the 1980s Japanese women have had the highest life expectancy in the world. In 1950 Denmark was among the European countries with highest life expectancies, but both Danish women and men have experienced relatively little progress since 1950. In 2010 French women had the highest life expectancy in Europe. While Japanese and French women have high life expectancies, the gender gap in these countries is relatively big (almost 7 years), whereas Denmark has a small gender gap (4 years).

### Fit of the model

**Fig. 3:**
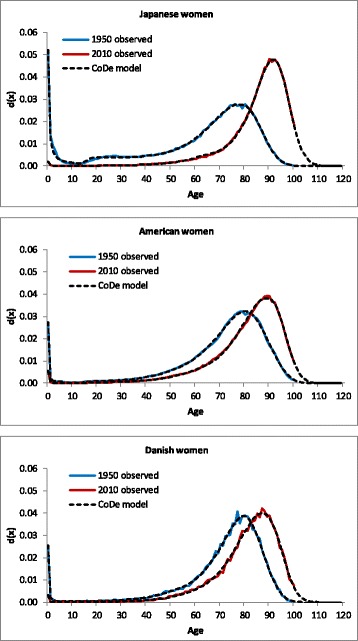
Age-at-death distribution, Japanese, American, and Danish women, 1950 and 2010, observed and fitted by the CoDe model

The CoDe model provides a good fit of the age-at-death distribution of not only French women, but also Japanese, US, and Danish women, both in 1950 and 2010 (see Fig. 3). Appendix 5 shows that the CoDe model provides a good fit of the age distribution for men in these four countries in 1950 and 2010 as well. For the years in between, the model fits the data well too. The average value of R^2^ across all years in the period 1950–2010 equals 99.8% for the age-at-death distribution and 99.7% for the logarithm of the age-specific death probabilities. On average the R^2^s for women and men are equal. The minimum value of R^2^ equals 99.3% for the age-at-death distribution and 98.4% for the log death probabilities. In both cases the minimum values are measured for Denmark. This can be explained by the fact that the age pattern of mortality for Denmark shows relatively large random fluctuations due to the fact that the Danish population is considerably smaller than the population of the other three countries.

### Time trends in the parameters

The changes in the values of the parameters of the model explain both the shift of the distribution to the right and the change in the shape of the distribution. Figure 3 shows that particularly for Japanese women the modal age at death has risen strongly, while the age-at-death distribution has become much more compressed. In contrast, in Denmark there has hardly been compression.

**Fig. 4:**
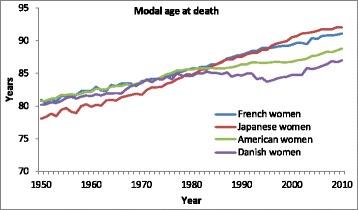
Modal age at death, French, Japanese, American, and Danish women, 1950–2010

The CoDe model can be used to disentangle the effects of delay and compression of mortality on changes in the age-at-death distribution by assessing the effects of changes in the estimated parameter values on the distribution. Figure 4 shows the change in the modal age at death from 1950 to 2010 for French, Japanese, American, and Danish women. The linear increase in the modal age for French and Japanese women shows that there has been a continuous shift of the distribution during the last 60 years. Since 1950 mortality of Japanese women has been delayed by 2.3 years per decade on average and for French women by 1.7 years. For American women the increase in the modal age slowed down in the 1980s, while for Danish women the 1980s even showed a decrease in the modal age at death. Since 2000 the modal age for American and Danish women has increased slightly more than for the other two countries.

**Fig. 5:**
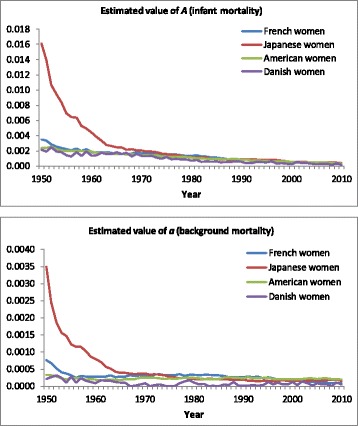
Estimated values of *A* (level of infant mortality) and *a* (background mortality), French, Japanese, American, and Danish women, 1950–2010

Changes in the shape of the age-at-death distribution can be explained by changes in the estimated values of *A* and *a*, i.e., changes in infant and adolescent mortality, respectively; the values of *b*_*1*_ and *b*_*2*_, i.e., changes in mortality at young adult and late middle age; and changes in *b*_*3*_, i.e., changes in mortality above the modal age. Figure 5 shows the estimated values of *A* and *a*. In the 1950s mortality in infancy and adolescence among Japanese women was considerably higher than in the other three countries. During the 1950s and 1960s there was a steep decline, followed by a gradual decline since the 1970s. During the last 40 years the differences across these four countries have been small.

**Fig. 6:**
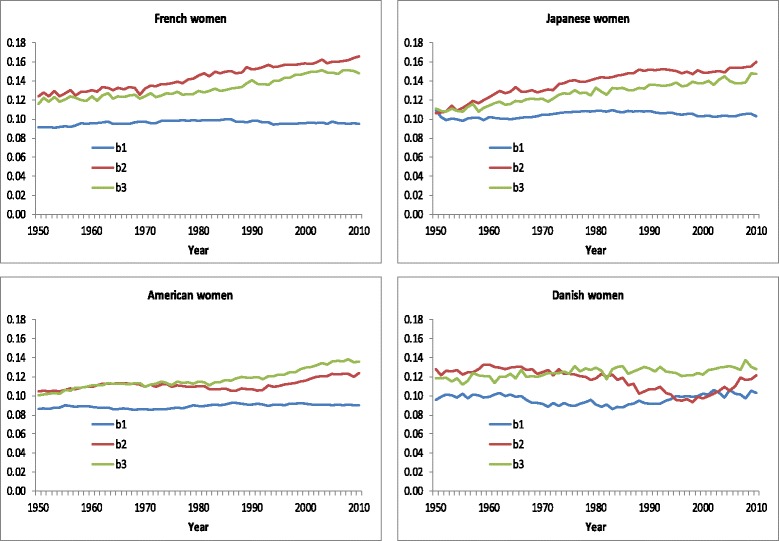
Estimated values of parameters *b*_*1*_, *b*_*2*_, and *b*_*3*_, French, Japanese, American, and Danish women, 1950–2010

Figure 6 shows the estimated values of *b*_*1*_, *b*_*2*_, and *b*_*3*_. For women in all four countries the value of *b*_*1*_ reflecting mortality until middle age has hardly changed. This confirms that we can assume *b*_*1*_ to be fixed across time when explaining changes in mortality. For French and Japanese women the value of *b*_*2*_ has shown a steady increase since 1950. This implies that there has been compression of mortality in late middle age as a higher value of *b*_*2*_ implies fewer deaths in middle age and more deaths around the modal age at death. For American women the estimated value of *b*_*2*_ did not increase before 1990. This implies that the decline in death probabilities among middle-aged American women between 1950 and 1990 can be explained fully by the delay of mortality. The increase in the value of *b*_*3*_ indicates compression of mortality above modal age, i.e., less decrease of mortality in advanced age than around the modal age at death. For Danish women the value of *b*_*3*_ was about constant, while the value of *b*_*2*_ decreased until 2000. This indicates that mortality among middle-aged Danish women developed less favorably than among older Danish women. Figure 16 in Appendix 6 shows the estimated values of *b*_*1*_, *b*_*2*_, and *b*_*3*_ for men. For Japanese and Danish men the values of *b*_*2*_ and *b*_*3*_ do not differ much, and a simpler version of the CoDe model without the fifth term of equation (2) would be sufficient to describe changes in the age pattern of mortality. In contrast, for French and American men, changes in *b*_*2*_ and *b*_*3*_ have been different since 1980. In these cases compression below and above the modal age do not run parallel and thus distinguishing *b*_*2*_ and *b*_*3*_ is needed for an accurate assessment of compression of mortality below and above the modal age.

### Effects of changes in the parameters on the age-at-death distribution

**Fig. 7:**
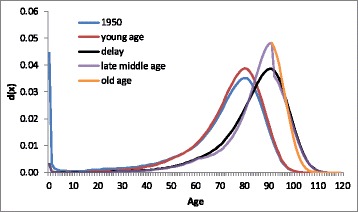
Effects of delay of mortality and changes in mortality in young, late middle, and old age between 1950 and 2010 on the age-at-death distribution, French women

Changes in the values of the parameters of the CoDe model make it possible to assess the effects of changes in mortality at different ages on the age-at-death distribution. For example, replacing the value of *A* estimated for 1950 by the value of *A* estimated for 2010 while maintaining the values estimated for 1950 for the other parameters in equation (2) we can calculate the death probabilities that would have resulted if the value of *A* only had changed between 1950 and 2010 and using a life table we can calculate the age-at-death distribution. Figure 7 shows the successive effects of changes in the parameter values on the age-at-death distribution for French women. The blue line shows the distribution for 1950, fitted by the CoDe model using the values of the parameters estimated for 1950. The red line shows how the distribution would have changed if only the values of *A* (infant mortality) and *a* (background mortality) had changed between 1950 and 2010. The red line shows that the decrease of mortality in young ages has led to compression, i.e., more deaths around the modal age. The black line shows the distribution that would have resulted if in addition to the changes in young ages the modal age at death had changed, but not the values of the *b*s. The increase in the modal age at death between 1950 and 2010 by 10 years has resulted in a shift of the distribution to the right. Comparing the purple with the black line shows how the increase in the value of *b*_*2*_ has resulted in compression due to fewer deaths in late middle age (around age 70) and more deaths around the modal age (90 years). Comparing the orange line with the black line shows how the increase in *b*_*3*_ reflects compression above the modal age at death: fewer deaths in old age (ages 97 and over) and more deaths around the modal age.

**Fig. 8:**
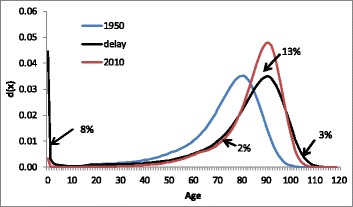
Fitted age-at-death distribution, French women, 1950 and 2010, and a simulated distribution that shows the effect of delay between 1950 and 2010 without a change in the shape of the distribution

**Fig. 9:**
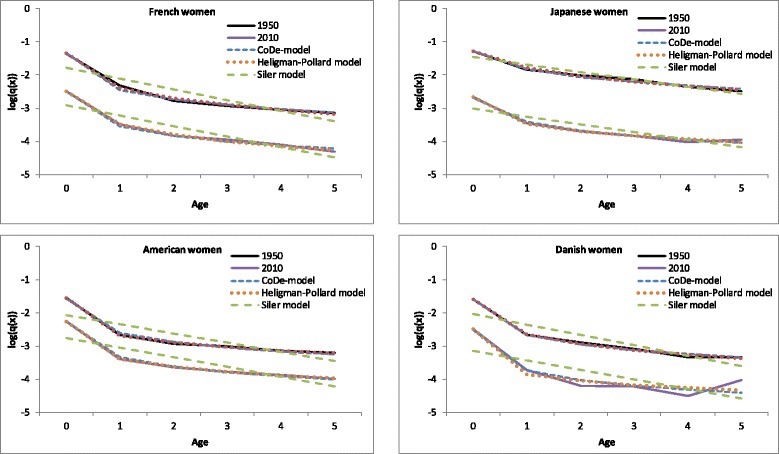
Logarithm of death probabilities, ages 0 to 5, French, Japanese, American, and Danish women, 1950 and 2010, observed and fitted by CoDe, Heligman-Pollard, and Siler models

**Fig. 10:**
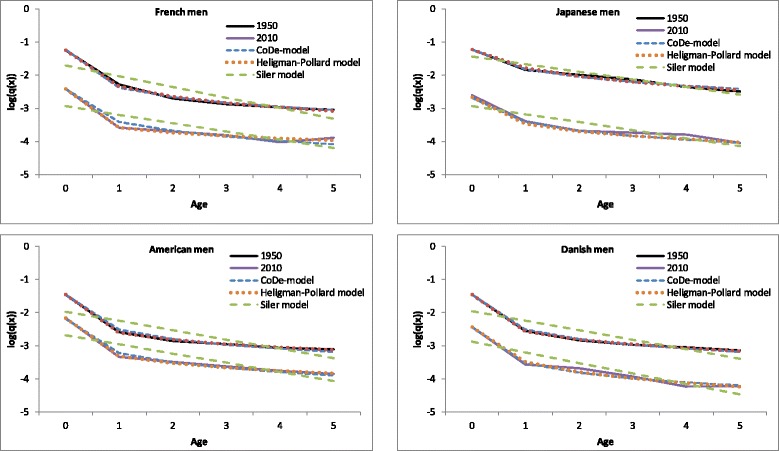
Logarithm of death probabilities, ages 0 to 5, French, Japanese, American, and Danish men, 1950 and 2010, observed and fitted by CoDe, Heligman-Pollard, and Siler models

**Fig. 11:**
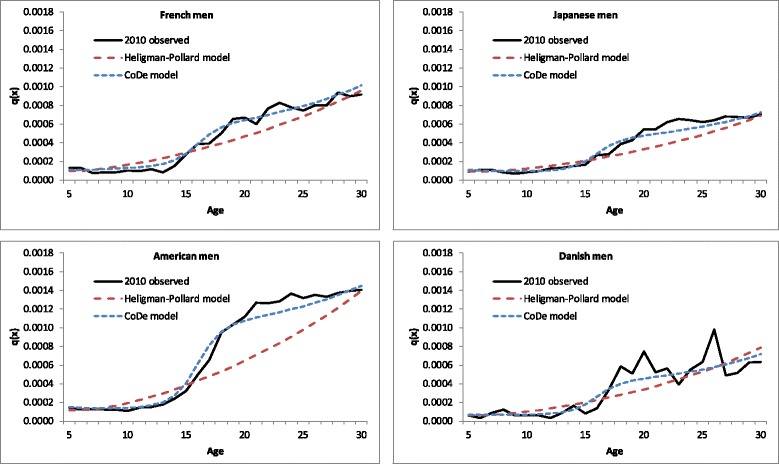
Death probabilities ages 5 to 30, French, Japanese, American, and Danish men, 2010, observed and fitted by the CoDe model and the Heligman-Pollard model

**Fig. 14:**
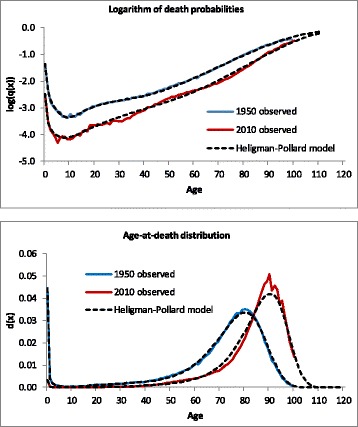
Logarithm of death probabilities and age-at-death distribution, French women, 1950 and 2010, observed and fitted by the Heligman-Pollard model

**Fig. 16:**
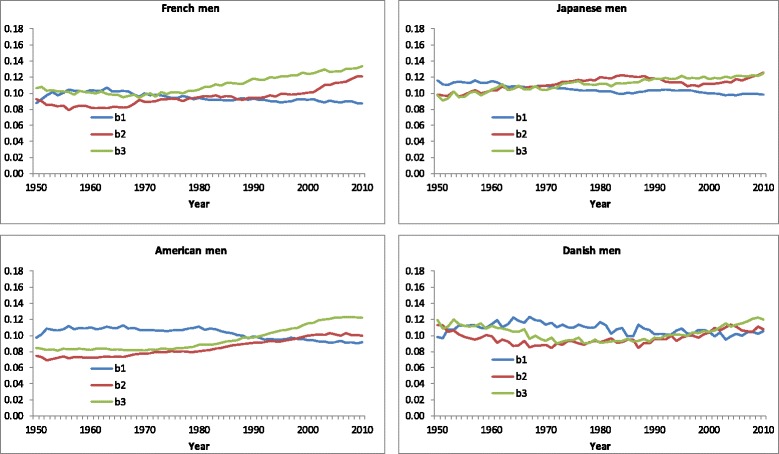
Estimated values of parameters *b*_*1*_, *b*_*2*_, and *b*_*3*_, French, Japanese, American, and Danish men, 1950–2010

In order to assess the total effect of compression in young and old ages on the shape of the age-of-death distribution for French women, Fig. 8 compares the fitted distribution for 2010 (red line) with the distribution that would have resulted from a shift of the 1950 distribution without a change in the shape (black line). Compression leads to an increase in the proportion of deaths around the modal age at death which can be measured by the surface between the red and black lines around the modal age. This increase equals 13%. The main part of this increase (8%) is due to fewer deaths in young age, while 2% is due to fewer death among women in their 70s and 3% is due to fewer deaths in old age. Thus compression of mortality in young ages has had a bigger impact on the age-at-death distribution than compression in old age. Appendix 7 shows the effects of compression on the age-at-death distribution for French men, and Japanese, American, and Danish women and men.

### Decomposing the change in life expectancy into delay versus compression at different life stages

From the effects of the changes in the parameter values on the life table survival rates, we can calculate the effects on the changes in life expectancy at birth. For this purpose we calculated life expectancies at birth for each of the simulated age-at-death distributions shown in Fig. 7. The difference between the life expectancies corresponding with the red and blue lines provides an estimate of the effect of changes in mortality in young ages on life expectancy at birth. The difference between life expectancies corresponding with the black and red lines estimates the effect of delay on life expectancy. Accordingly, the effects of changes in mortality in late middle age and old age on life expectancy are estimated. Table 1 shows the contribution of the delay in mortality, i.e., shift of the age-at-death distribution and differences in the changes in mortality in young (*A* + *a*), adult (*b*_*1*_ + *b*_*2*_), and old age (*b*_*3*_), i.e., changes in the shape of the distribution to changes in life expectancy at birth between 1950 and 2010 based on data for 1950 and 2010. Because life expectancy estimated from the age-at-death distribution fitted by the CoDe model differs slightly from observed life expectancy in both 1950 and 2010, part of the observed changes between 1950 and 2010 are not explained by the CoDe model, and are labeled as “unexplained” in the table.

**Table 1:** Effect of changes in values of parameters of the CoDe model on change in life expectancy at birth between 1950 and 2010

	France		Japan		US		Denmark	
	women	men	women	men	women	men	women	men
Total change in life expectancy at birth due to	15.51	14.61	25.43	21.97	10.19	10.96	9.80	8.01
delay of mortality (changed in M)	9.87	10.42	13.80	12.12	7.85	10.35	6.76	4.12
changed in mortality								
in young age (change in A and a)	5.05	5.21	12.10	11.73	2.38	2.92	2.45	3.48
in middle age (change in b1 and b2)	0.77	−0.25	−0.39	−1.57	0.43	−1.30	0.70	0.65
above modal age at death (change in b3)	−0.44	−0.43	−0.52	−0.48	−0.42	−0.79	0.00	0.05
unexplained	0.26	−0.35	0.45	0.16	−0.07	−0.21	−0.11	−0.29

Table 1 shows that in France life expectancy at birth has increased by some 15 years since 1950 and that two-thirds of this increase can be attributed to the delay of mortality, while the decline of mortality in young age contributed some 5 years to the increase in life expectancy. In Japan the decline in mortality in young ages has caused about half of the total increase in life expectancy. Note that the levels of infant and background mortality in Japan in 1950 were considerably higher than in the other three countries. Since about 1980 the differences across the four countries have been small and changes in mortality in young ages have had similar impacts on the increase in life expectancy (see Appendix 9). Figure 4 showed a continuous increase in the modal age for French and Japanese women since 1950, but a stagnation in the increase of the modal age after 1980 for American and Danish women. This is reflected by the smaller impact of the delay of mortality on life expectancy in the US and Denmark.

Changes in the shape of the age-at-death distribution in adult and old age have had a much smaller impact on life expectancy than the shift of the distribution due to delay of mortality.

Changes in mortality in middle age have had a negative impact on life expectancy for men in all countries but Denmark. Note that this does not imply that mortality in middle age has increased, but rather that mortality in middle age has declined less strongly than around the modal age at death. Thus there has been no compression of mortality between middle age and the modal age.

The impact of changes in mortality in old age has been negative. This implies that there has been compression of mortality in old age. Note that compression in old age has a negative impact on life expectancy in contrast with compression below the modal age at death.

In sum, since 1950 life expectancy has mainly increased due to a shift in the age-at-death distribution. Two-thirds of the increase in life expectancy can be attributed to the delay of mortality. If we add the effects of changes in young and middle age we observe that compression of mortality below the modal age has contributed about one-third to the increase in life expectancy. Compression of mortality above the modal age has had a small negative effect.

## Discussion

The results of the CoDe model show that two-third of the increase in life expectancy at birth between 1950 and 2010 in Japan, France, the US, and Denmark can be attributed to the shift in the mortality age schedule. This finding of postponement of mortality having had a much bigger effect than compression is in line with the recent results by Janssen et al. [45], who performed a decomposition of life expectancy at age 50 into the effect of compression versus delay in nine European countries between 1950 and 2009, using the decomposition method by Rossi et al. [20]. Bergeron-Boucher et al. – very recently – found for Swedish females as well that since the mid-1960s more than 70% of the increase in life expectancy at birth was caused by delay [21]. However, they did not distinguish between compression at different stages of life.

Compression can explain the remaining one-third of the increase in life expectancy at birth between 1950 and 2010 in the four countries we studied. We distinguished in our model compression of mortality at different ages. That is, changes in infant mortality (*A*), and changes in both teenage excess mortality and the level of background mortality *(a)* together describe the compression of mortality at young ages, whereas changes in the value of *b*_*2*_ describe the effect of compression of mortality in late middle age and *b*_*3*_ describes the effect of compression in old age.

The empirical results showed that it is indeed useful to make this distinction. Over the period 1950–2010, compression of mortality due to the decline in mortality in young ages was most important and caused one-third of the increase in life expectancy. In four of the eight populations we observed compression in middle age, but in the other four populations mortality in middle age declined less strongly than around modal age. Compression of mortality above the modal age at death, resulting from a smaller decline in mortality in old age compared to adult ages, had a slight negative impact on life expectancy.

While the estimated value of *b*_*1*_ has hardly changed since 1950 and has not differed much across countries, the values of *b*_*2*_ and *b*_*3*_ have shown variation across time and across countries. The estimated value of *b*_*1*_ has been close to 0.10 in most countries. This is consistent with Thatcher’s finding that from soon after 30 years of age the probability of dying starts to rise by about 10% with each successive year of age [4]. Also it corresponds to Bongaarts’ finding that the slope parameter of the logistic model is nearly constant over time [12]. The variation in the changes of the values of *b*_*2*_ and *b*_3_ shows that while a simple model including *b*_*1*_ only is capable of describing the age pattern of mortality in adult ages in 1950, an accurate description of current changes in mortality in middle and advanced age requires additional *b* parameters.

## Conclusions

In this article, we presented a new model to describe the full age pattern of mortality and to assess delay and compression of mortality at different stages in life. Our results show that the CoDe model provides an accurate description of the entire age pattern of mortality in four low-mortality countries: Japan, France, the US, and Denmark. Changes in the values of five parameters adequately describe changes in both death probabilities and the age-at-death distribution between 1950 and 2010.

When compared with other parametric mortality models, the CoDe model has several advantages. First, the CoDe model uses fewer parameters to describe infant and childhood mortality than the Heligman-Pollard model and provides a better fit than the exponential model suggested by Siler [16] and others. The CoDe model takes account of changes through time in infant and childhood mortality by changes in the value of one parameter, whereas the Heligman-Pollard model includes three parameters which are highly correlated. Second, the CoDe model uses fewer parameters than the Heligman-Pollard and Rogers-Little models to describe the excess mortality in adolescence. One parameter is sufficient to explain changes through time. Third, the CoDe model includes a logistic model to describe mortality in adolescence which implies a constant level beyond early adulthood. Thus the CoDe model takes account of background mortality, without requiring an additional parameter. Fourth, the CoDe model includes one parameter to describe shifts in adult and old age mortality. As this parameter equals the modal age at death, it is straightforward to interpret changes in its value. Fifth, the CoDe model distinguishes three separate parameters to account for the increase in mortality by age in adult and advanced age. This provides a better fit of mortality in old age. Whereas the increase of mortality by age in young adult ages has not changed over time, the increase in middle and old age has. Changes in the value of the parameter describing the increase in mortality by age above the modal age at death accounts for compression of mortality in advanced age. Sixth, the CoDe model provides an accurate fit of both the age schedule of death probabilities and the age-at-death distribution. Seventh, changes in mortality over time can be explained by changes in the values of five parameters which reflect changes in mortality in successive stages of life. Changes in these parameters allow us to assess which part of the increase in life expectancy can be attributed to delay of mortality and which part to compression.

For specific purposes the CoDe model may be simplified. For example, if one is interested in delay and compression without making a distinction between compression in young, middle or old age, the CoDe model can be reduced from five to three terms, i.e., if one assumes that *b*_*2*_ = *b*_*3*_ = *b*_*1*_ the last two terms of equation (2) can be deleted. In this case, compression of mortality in adult age is described by changes in the value of *b*_*1*_. Alternatively, if one is interested in mortality in adult age only, the first term of the model describing infant mortality can be deleted and the second term can be reduced to a constant describing background mortality.

The CoDe model provides a powerful tool for describing current changes in mortality and gives insight into the determinants. Our findings, however, also have implications for forecasting future changes in mortality. Demographers agree that in low-mortality countries the levels of infant and background mortality are so low that no big further declines may be expected [33]. This implies that future increases in life expectancy will depend on further shifts in the mortality age schedules. Our results show that between 1950 and 2010 the age schedules have shifted by some 2 months per year. Since mortality rates in old age have declined less strongly than mortality rates around the modal age at death, life expectancy at birth has increased slightly less strongly than the shift of the survival curve, but the effect of compression of mortality in old age has been relatively small between 1950 and 2010. If the delay of mortality continues and if the effect of compression in old age does not become much stronger in the future, a substantial further increase in life expectancy can be projected.

## Figures and Tables

**Fig. 12:**
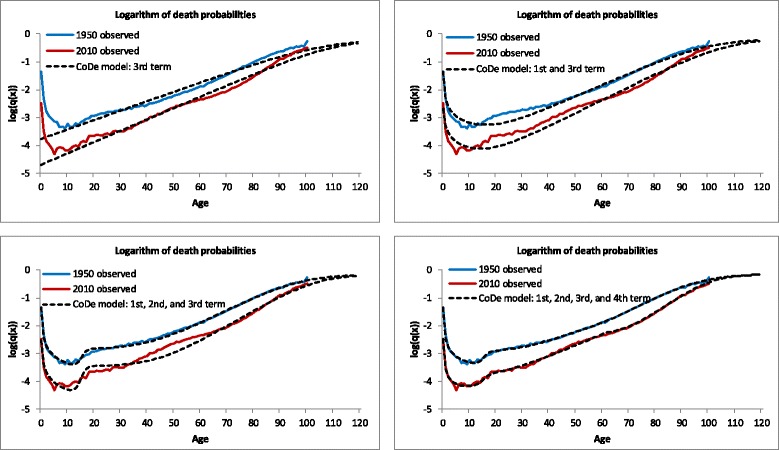
Logarithm of death probabilities, French women, 1950 and 2010, four variants of the CoDe model

**Fig. 13:**
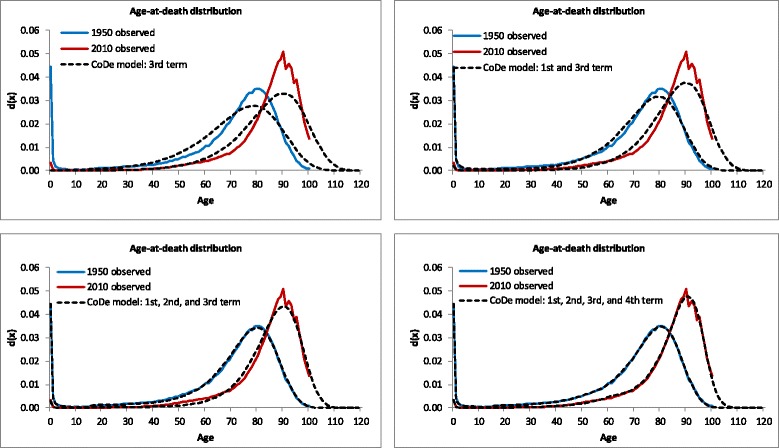
Age-at-death distribution, French women, 1950 and 2010, four variants of the CoDe model

**Fig. 15:**
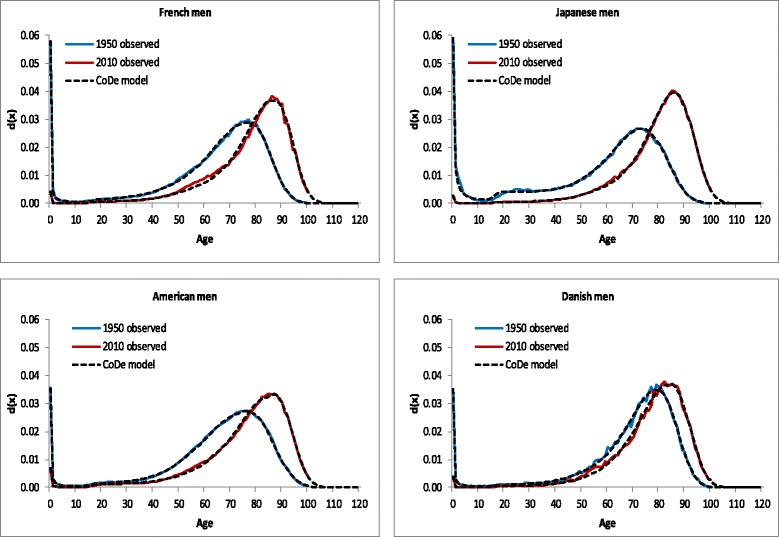
Age-at-death distribution, French, Japanese, American, and Danish men, 1950 and 2010, observed and fitted by the CoDe model

**Fig. 17:**
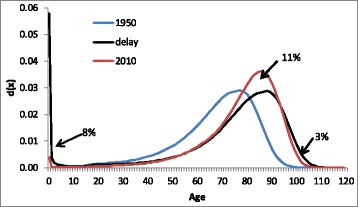
Fitted age-at-death distribution, French men, 1950 and 2010, and a simulated distribution that shows the effect of delay between 1950 and 2010 without a change in the shape of the distribution

**Fig. 18:**
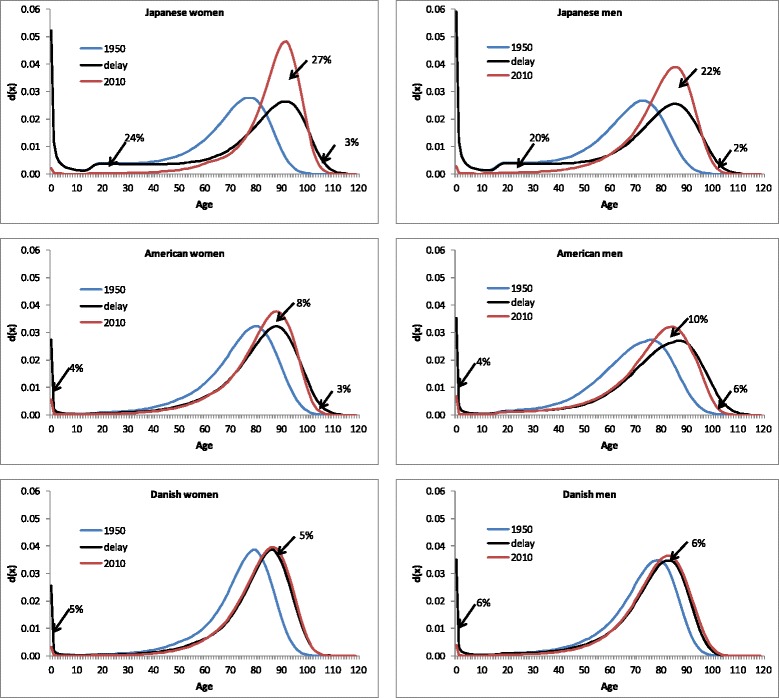
Fitted age-at-death distribution, Japanese, American, and Danish women and men, 1950 and 2010, and simulated distributions that show the effect of delay between 1950 and 2010 without a change in the shape of the distribution

## Appendix 1

### Comparison of the fit of the CoDe, Heligman-Pollard and Siler model to under-5 mortality

The CoDe model describes death probabilities in infancy by a term including two parameters *A*/(*x* + *B*). The Siler model uses two parameters for mortality in infancy as well: *a*_*1*_ exp(−*a*_*2*_ *x*). The Heligman-Pollard model uses three parameters: ${A}^{x+{B}^c}$. In order to compare the fit of the three models for mortality in infancy we estimated the parameters of each model by minimizing the MSE of the logarithm of death probabilities from age 0 to 5. Figure 9 shows the fit of the three models for French, Japanese, American, and Danish women in 1950 and 2010. The fit of the CoDe model and the Heligman-Pollard model does not differ much. The Heligman-Pollard model is slightly more accurate, but at the cost of using one additional parameter. The fit of the Siler model is clearly less accurate than that of the other two models. Figure 10 shows that the results for men are comparable to those for women. The Heligman-Pollard model produces a slightly better fit than the CoDe model, since for French and American men the CoDe model overestimates death probabilities at age 1.

## Appendix 2

### Comparison of the fit of the CoDe model and the Heligman-Pollard model in adolescence

The CoDe model uses a simple logistic model to describe death probabilities in adolescence. This model describes the increase in death probabilities around age 16, which is often called the “teenage hump.” The teenage hump is a male phenomenon. Figure 11 shows that the CoDe model provides a better fit of mortality between ages 5 and 30 for French, Japanese, American, and Danish men in 2010 than the Heligman-Pollard model. Even though the logistic model used to describe the teenage hump implies a constant level of death probabilities in adult age, reflecting background mortality, Fig. 11 shows an increase of mortality of men in their twenties. This is explained by the third term of equation (2) describing the increase in adult mortality by age.

## Appendix 3

### Contribution of the separate terms of the CoDe model on the fit of the model

The CoDe model includes five terms (see Eq. (2)), which represent mortality in infancy, adolescence, early adulthood, late adulthood, and old age. Figures 12 and 13 show the contribution of each term to the fit of the CoDe model for French women in 1950 and 2010.

We start with the third term of equation (2), i.e., a logistic function. This comes down to assuming that *A* = *a* = 0 and *b*_*2*_ = *b*_*3*_ = *b*_*1*_. The upper left part of Fig. 12 shows that the logistic model fits the average increase of death probabilities in adult age. However, since this model overestimates death probabilities below the modal age at death, it underestimates the number of deaths around the modal age at death (see Fig. 13). The *R* ^*2*^ for the fit of the age-at-death distribution equals 78.3% for 1950 and 90.6% for 2010 and for the logarithm of the death probabilities 86.3% for 1950 and 92.2% for 2010.

When we add the first term, representing mortality in infancy, the fit of the model improves in young age, but it also leads to a better fit of mortality in adult age, as is shown by the upper right part of Fig. 12. This is due to the fact that the estimated value of *b*_*1*_ in the model, including a term of mortality in infancy, exceeds the estimated value of *b*_*1*_ for the model including the third term alone. By adding the first term the value of *R* ^*2*^ for the age-at-death distribution increases from 78.3 to 98.5% in 1950 and from 90.6 to 95.3% for 2010. For the logarithm of the death probabilities *R* ^*2*^ increases from 86.3 to 97.5% and from 92.2 to 96.7% for 2010.

Adding the second term, representing the teenage hump and background mortality, has two effects. First, it produces a better fit of mortality in adolescence. Second, it results in a further increase of the estimated value of *b*_*1*_. As a result the estimate of death probabilities in the years before the modal age improves. See the lower left part of Fig. 12. This results in an improvement of the fit of the age-at-death distribution around the modal age, as can be seen from the lower left part of Fig. 13. Adding the second term leads to an increase in the value of *R* ^*2*^ for the age-at-death distribution from 98.5 to 99.8% in 1950 and from 95.3 to 98.6% in 2010 and an improvement of the of *R* ^*2*^ for the logarithm of the death probabilities from 97.5 to 99.6% in 1950 and from 96.7 in to 97.8% in 2010.

Adding the fourth term allows to make a distinction between mortality in early and late adulthood. The lower right part of Fig. 12 shows that this has a relatively small impact on the fit of the model for 1950, but leads to a considerable improvement of the fit in young adult age for 2010. Figure 13 shows that this leads to a better estimate of the age-at-death distribution for 2010. Thus a model that does not distinguish between mortality in early and late adulthood would lead to underestimating compression of mortality between 1950 and 2010. The value of *R* ^*2*^ improves from 99.8 to 99.9% for the age-at-death distribution in 1950 and from 98.6 to 99.7% for 2010. For the logarithm of the death probabilities the value of *R* ^*2*^increases from 99.6 to 99.8% in 1950 and from 97.8 to 99.8% in 2010.

Adding the fifth term, representing mortality above the modal age at death, does not lead to much further improvement of the fit, as can be seen from comparing the lower right parts of Figs 12 and 13 with Fig. 2. The main reason for including the fifth term is not that this improves the fit of the model but that we want to distinguish the impact of changes in mortality above the modal age at death on compression from the effect of changes below the modal age. The reason that this distinction is useful is that compression below the modal age at death has a positive impact on life expectancy, while compression above the modal age has a negative impact. Compression above the modal age can be an indication that we are approaching a limit to the increase in the average lifespan.

## Appendix 4

### Fit of the Heligman-Pollard model, French women, 2010

Figure 14 shows the fit of the Heligman-Pollard model [17] to age-specific death probabilities and the age-at-death distribution for French women in 1950 and 2010. The Heligman-Pollard model produces an accurate fit to the data for 1950. However for 2010 the fit of the age-at-death distribution is less accurate than that of the CoDe model as shown in Fig. 14. The value of *R* ^*2*^ for the fit of the Heligman-Pollard model to the age-at-death distribution in 2010 equals 98.3%, compared with 99.7% for the CoDe model. The Heligman-Pollard model overestimates the proportion of deaths between ages 70 and 80 and underestimates deaths around the modal age. In addition the Heligman-Pollard model overestimates death probabilities in young adult age in 2010. Thus the Heligman-Pollard model underestimates compression of mortality in 2010. The CoDe model produces a better fit than the Heligman-Pollard model for Japan, the US, and Denmark as well, both for men and women (see Table 2). For 1950 the differences between both models are very small, but for 2010 the fit of the CoDe model clearly is better than that of the Heligman-Pollard model.

**Table 2:** Comparison of the fit of the CoDe model and the Heligman-Pollard model measured by weighted mean squared error (MSE), France, Japan, US, and Denmark, 1950 and 2010

		MSE (×1000)			
		1950		2010	
		CoDe model	Heligman-Pollard model	CoDe model	Heligman-Pollard model
France	women	1.00	1.93	2.30	11.47
	men	1.42	1.85	5.24	11.09
Japan	women	4.60	3.07	0.94	13.64
	men	5.17	5.53	1.30	4.66
US	women	0.92	1.72	0.65	2.43
	men	1.83	3.39	1.71	6.36
Denmark	women	3.83	4.82	9.62	10.93
	men	4.86	5.29	6.43	9.15
Average		2.95	3.45	3.52	8.72

## Appendix 5

### Age-at-death distribution, French, Japanese, American, and Danish men, 1950 and 2010

Figure 15 shows that the CoDe Model produces an accurate fit of the age-at-death distribution for French, Japanese, American, and Danish men in both 1950 and 2010. The goodness of fit is comparable to that for women as shown in Figs 2 and 3. The fit is accurate for the other years in the period 1950–2010 as well. For the age-at-death distribution the value of *R* ^*2*^ exceeds 99.4% in every single year. For the logarithm of the death probabilities the value of *R* ^*2*^ exceeds 99.5% for French and American men in each year. For Japanese men *R* ^*2*^ exceeds 99.5% in all but 1 year and for Danish men *R* ^*2*^ exceeds 99.0% in all but 4 years. One main reason for the slightly less accurate fit for Danish men is that the observations show more irregular fluctuations than for the other countries due to smaller population numbers.

## Appendix 6

### Time trends of the estimated values of *b*_*1*_, *b*_*2*_, and *b*_*3*_ for men

Figure 16 shows that from 1950 to 2010 for French, Japanese, American, and Danish men the value of *b*_*1*_ has fluctuated around a value of 0.10. For Japanese and Danish men the values of *b*_*2*_ and *b*_*3*_ do not differ much. In contrast, for French and American men the value of *b*_*3*_ has exceeded that of *b*_*2*_. For American men the value of *b*_*3*_ has increased more strongly than that of *b*_*2*_ since 1980.

## Appendix 7

### Impact of compression of mortality between 1950 and 2010 on age-at-death distribution, French men, and Japanese, American, and Danish women and men

A comparison of Fig. 17 with Fig. 8 shows that the age-at-death distribution for French men in both 1950 and 2010 is less compressed than the age-at-death distribution for French women. Since 1950 there has been slightly less compression of mortality for French men than for women. If we compare the distribution for 2010 for French men with the distribution that would have occurred if there would have been delay of mortality without a change in the shape of the distribution, the proportion of deaths around the modal age in 2010 was 11% higher compared with 13% higher for French women as shown in Fig. 8. The difference is due to fewer deaths among French women in their 70s.

Figure 18 shows considerable differences in compression of mortality in Japan, the US, and Denmark. In Japan the shape of the age-at-death distribution has changed considerably due to a strong decline of mortality in young ages. The proportion of deaths around the modal age in 2010 was about a quarter higher than in 1950. In France and Japan there has been more compression than in the US and Denmark. Among American men there has been more compression of mortality in old age than among American women. In Denmark the shape of the age-at-death distribution has hardly changed, apart from a decrease of mortality in infancy.

## Appendix 8

### Impact of the choice of the asymptotic value of death probabilities on the assessment of the effects of delay and compression

One parameter in the CoDe model (formula 2) is the asymptotic value of death probabilities, *g*. The value of this parameter affects the estimated value of the parameter *b*_*3*_. In order to make estimates of *b*_*3*_ comparable across years and across countries we decided to assume *g* to remain constant across years and equal across countries. Gampe [42] suggests that in very old age death probabilities move to a value of 0.5. We examined whether a value of *g* = 0.5 produces the best fit. It turned out that for the four countries in this study *g* = 0.7 produces a slightly better fit for men and about equal fit for women. (See Table 3). For this reason we decided to estimate the parameters of the CoDe model assuming that *g* = 0.7.

**Table 3:** Fit of the CoDe model measured by weighted mean square error (MSE), assuming deifferent asymptotic value (g) of death probabilities

		MSE (×1000)	
		*g* = 0.5	*g* = 0.7
France	women	1.31	1.34
	men	2.58	2.59
Japan	women	1.14	1.16
	men	1.79	1.63
US	women	1.22	1.20
	men	1.87	1.73
Denmark	women	5.63	5.62
	men	5.28	5.11
Average	women	2.32	2.33
	men	2.88	2.77

Choosing another value of *g* affects the estimate of the value of *b*_*3*_. For women, assuming *g* = 0.5, the estimated value of *b*_*3*_ is about 0.02 higher on average in 2010 than when it is assumed that *g* = 0.7. However, as the value of *b*_*3*_ is higher in all years, it hardly affects the estimated change in the value of *b*_*3*_ between 1950 and 2010 and thus hardly affects the assessment of the effects of compression in old age on the change in life expectancy, as is shown by Table 4: the results hardly differ from those in Table 1. For men the differences between Tables 1 and 4 are small as well.

**Table 4:** Effect of changes in values of parameters of the CoDe model on change in life expectancy at birth between 1950 and 2010. Assuming *g* = 0.5

	France		Japan		US		Denmark	
	women	men	women	men	women	men	women	men
Total change in life expectancy at birth due to	15.51	14.61	25.43	21.97	10.19	10.96	9.80	8.01
delay of mortality (changed in M)	9.99	10.42	13.80	12.12	7.80	10.35	6.87	4.28
changed in mortality								
in young age (change in A and a)	5.05	5.24	12.19	11.77	2.39	2.92	2.45	3.48
in middle age (change in b1 and b2)	−0.75	−0.26	−0.40	−1.52	−0.50	−1.26	0.62	0.56
above modal age at death (change in b3)	−0.38	−0.45	−0.55	−0.49	−0.42	−0.78	0.01	0.03
unexplained	0.10	−0.35	0.39	0.09	−0.08	−0.27	−0.14	−0.34

## Appendix 9

### Effects of delay and compression on changes in life expectancy at birth between 1950 and 1980 and between 1980 and 2010

Table 5 shows that the increase in life expectancy at birth of women has slowed down since 1980. The reason is that the effect of the decline in mortality in young age between 1950 and 1980 was much bigger than between 1980 and 2010. In addition, delay has slowed down since 1980 in the US and Denmark. For men, life expectancy at birth has increased more strongly between 1980 and 2010 than between 1950 and 1980 in France, the US, and Denmark. This was caused by more delay. In Japan the effect of the decrease of mortality in young ages exceeded the effect of delay. Since 1980 average delay has been 0.2 years or 2 months per year.

**Table 5:** Effects of changes in values of parameters of the CoDe model on change in life expectancy at birth between 1950 and 1980, and between 1980 and 2010

	France		Japan		US		Denmark	
	women	men	women	men	women	men	women	men
1950–1980								
Total change in life expectancy at birth due to	9.22	6.73	17.84	15.79	6.45	4.58	5.66	2.07
delay of mortality (changed in M)	4.81	2.57	6.54	6.37	4.62	2.73	4.33	−1.78
changed in mortality								
in young age	3.93	3.48	11.48	10.75	1.68	1.15	1.90	1.75
in middle age	0.69	0.62	−0.04	−1.15	0.37	0.88	−0.48	1.53
above modal age at death	−0.13	0.00	−0.44	−0.36	−0.18	−0.15	−0.01	0.61
unexplained	−0.08	0.05	0.29	0.18	−0.04	−0.02	−0.07	−0.05
1980–2010								
Total change in life expectancy at birth due to	6.30	7.88	7.59	6.18	3.73	6.38	4.14	5.94
delay of mortality (changed in M)	4.94	7.74	7.19	5.61	3.18	7.49	2.38	5.99
changed in mortality								
in young age	1.25	1.88	0.68	1.08	0.76	1.93	0.59	1.75
in middle age	0.07	−0.91	−0.29	−0.37	0.05	−2.19	1.20	−1.07
above modal age at death	−0.31	−0.43	−0.15	−0.13	−0.24	−0.66	0.00	−0.49
unexplained	0.34	−0.40	0.16	−0.01	−0.02	−0.19	−0.04	−0.24

Note that adding the estimates of the effects of delay and compression for the period between 1950 and 1980 and those for the period 1980 to 2010 yields almost the same results as the total effects for the period 1950 and 2010 shown in Table 1. The minor differences are due to the fact that even though the CoDe model fits the data very well, there are small differences between the actual and estimated life expectancy in 1980.

## Abbreviations

CoDe model: Compression and Delay of mortality model
HMD: Human Mortality Database
MSE: Mean squared error
